# Biological and Mechanical Characterization of the Random Positioning Machine (RPM) for Microgravity Simulations

**DOI:** 10.3390/life11111190

**Published:** 2021-11-05

**Authors:** Marco Calvaruso, Carmelo Militello, Luigi Minafra, Veronica La Regina, Filippo Torrisi, Gaia Pucci, Francesco P. Cammarata, Valentina Bravatà, Giusi I. Forte, Giorgio Russo

**Affiliations:** 1 Institute of Molecular Bioimaging and Physiology, National Research Council (IBFM-CNR), 90015 Cefalù, Italy; marco.calvaruso@ibfm.cnr.it (M.C.); carmelo.militello@ibfm.cnr.it (C.M.); francesco.cammarata@ibfm.cnr.it (F.P.C.); valentina.bravata@ibfm.cnr.it (V.B.); giusi.forte@ibfm.cnr.it (G.I.F.); giorgio-russo@cnr.it (G.R.); 2 Nanoracks Space Outpost Europe SRL, 10121 Torino, Italy; vlaregina@nanoracks.com; 3 Departments of Biomedical and BioTechnological Science (BIOMETEC), University of Catania, 95123 Catania, Italy; filippo.torrisi@unict.it; 4 Department of Biological, Chemical and Pharmaceutical Sciences and Technologies (STeBiCeF), University of Palermo, 90128 Palermo, Italy; gaia.pucci91@gmail.com

**Keywords:** space biology, cancer biology, TNBC, simulated microgravity, random positioning machine

## Abstract

The rapid improvement of space technologies is leading to the continuous increase of space missions that will soon bring humans back to the Moon and, in the coming future, toward longer interplanetary missions such as the one to Mars. The idea of living in space is charming and fascinating; however, the space environment is a harsh place to host human life and exposes the crew to many physical challenges. The absence of gravity experienced in space affects many aspects of human biology and can be reproduced in vitro with the help of microgravity simulators. Simulated microgravity (s-μg) is applied in many fields of research, ranging from cell biology to physics, including cancer biology. In our study, we aimed to characterize, at the biological and mechanical level, a Random Positioning Machine in order to simulate microgravity in an in vitro model of Triple-Negative Breast Cancer (TNBC). We investigated the effects played by s-μg by analyzing the change of expression of some genes that drive proliferation, survival, cell death, cancer stemness, and metastasis in the human MDA-MB-231 cell line. Besides the mechanical verification of the RPM used in our studies, our biological findings highlighted the impact of s-μg and its putative involvement in cancer progression.

## 1. Introduction

Since the first human Moon walk in July 1969, the interest towards space missions and human life in space has been increasing. The idea to create a space outpost for humans is no longer related to science fiction, and the discovery of lunar water by Chandrayaan-1 in 2008 [1], together with evidence of the presence of river and lake beds on Mars [2] has concretely opened the route for space colonization that, in the coming future, could be reasonably achievable. If, on one hand, space could offer a new shelter for humans concerned about their future life conditions on Earth, on the other it will expose space inhabitants to new physical challenges that may affect their healthy conditions [3].

The influence of microgravity on biological systems has been under analysis since the aftermath of the last human mission on the Moon, the Apollo 17. The Skylab Project, started in 1973, represents a milestone for all the experiments which studied the influence exerted by microgravity on biological systems and documented changes in several apparatuses of the human body and human cell lines [4,5]. The Skylab Project was followed, in 1981, by NASA’s Space Transportation System (STS), also known as the Space Shuttle Program, which ended in 2011. In a time span of 30 years, 135 missions were accomplished, with 36 aimed to transport, from Earth to orbit, all the components required to perform a huge array of experiments that covered many disciplines, from astrophysics to life science. Many studies were conducted inside the space shuttle, used as a unique laboratory, where the effects of real microgravity could be tested: cell and molecular biology, including immunology; developmental biology; plant biology; radiation biology; and animal and human physiology [6]. With the end of the Space Shuttle Program, studies in space are currently performed on the International Space Station (ISS).

Thus, both the Skylab and Spacelab programs established the way for the development of space biology, which should still be considered as a ”young field of science research”, aimed not only to discoveries that may facilitate human life in space, but also and not less important, to better elucidate certain phenomena when we analyze them in unusual conditions as the ones that occur during gravity unloading.

In this direction, to reproduce the absence of gravity on Earth, some devices have been developed in recent years. The Rotating Wall Vessel (RWV) and Clinostat are considered to be 2D microgravity simulators, while the Random Positioning Machine (RPM) and 3D Clinostat simulate weightlessness in a three dimensional pattern, thus representing a bench-top method to study microgravity effects. The RWV and the 2D Clinostat both recreate microgravity by rotating around their horizontal axis; they reproduce a constant vertical fall, avoiding sedimentation of the samples and randomizing the gravity vector. Moreover, RWV can provide oxygen to the rotating samples, contrary to the 2D Clinostat [7,8]. The RPM and the 3D Clinostat are usually and erroneously referred to as the same device and their rotation occurs both along the x and y axis. However, there is a difference among them which relies on the speed of rotation of the x axis with respect to the y axis. In fact, for the 3D Clinostat the two axes rotate at the same speed and thus create the same and redundant trajectory of rotation. On the contrary, the rotation for the RPM may occur at different and random speeds for the two axes [9].

An interesting and alternative device to simulate microgravity without rotation is the one exploiting magnetic levitation. The physical principle behind magnetic levitation is the counterbalancing of gravity force by applying an opposite magnetic field to nullify gravity [10,11].

The development and further implementation of microgravity simulators have given a great impulse to the collection of data aimed at studying the effects of gravity unloading in a multitude of fields of research. The application of simulated microgravity (s-μg) is used, for example, in biophysics to study protein crystallization [12], microbiology and virology [13,14], and human physiology and pathology. As seen in astronauts, a prolonged experience in microgravitational conditions (as the one occurring on ISS during space missions) associated with an uninterrupted exposure to cosmic radiation, have a strong impact on their health. The cardiovascular, endocrine, ocular, musculoskeletal, respiratory, and immune systems are all affected by the permanence in microgravity [15].

Nevertheless, since cancer research represents a prominent field of research, many scientific groups worldwide are studying the effects played by s-μg, in terms of cancer survival and progression, using in vitro models [16].

Our previous work highlighted the underlying mechanisms concerning cancer response to ionizing radiation. In this context, some of our studies were focused on Triple-Negative Breast Cancer (TNBC), using both in vitro and in vivo models [17,18].

**Figure 1:**
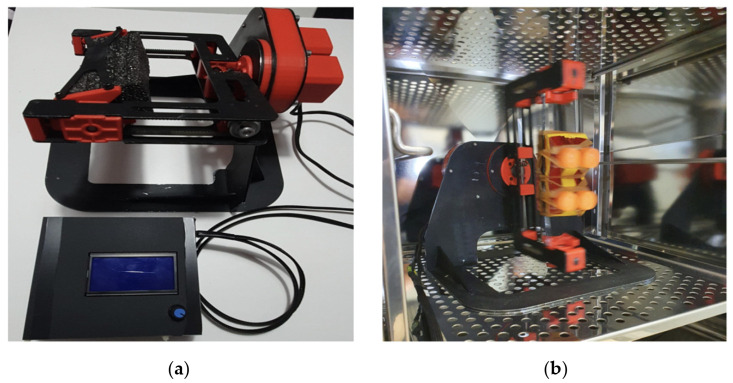
(**a**) The Random Positioning Machine (RPM) produced by AATC and (**b**) the RPM operating inside the cell incubator.

To analyze the s-μg influence on our in vitro model of TNBC, we have recently acquired a RPM (Figure 1) developed by the Analog Astronaut Training Center company (AATC, Kraków, Poland). For our study, we sought to determine the effects of microgravity using the TNBC cell model MDA-MB-231 previously characterized at the radiobiological level by our group [19]. TNBC is a highly aggressive and radioresistant form of breast cancer that account for 10–15% of breast tumors and characterized by a bad prognosis and a downward course. Because of the lack of specific tumor markers, patients cannot be treated with endocrine therapy or trastuzumab, and currently no exclusive strategies are available for TNBC forms. Therapy is mainly based on the administration of neoadjuvant and adjuvant chemotherapy, often associated with radiotherapy, but gold standard chemotherapy has not yet been established [20].

The aim of the present work is to characterize both biologically and mechanically the RPM, in order to collect data demonstrating the machine efficiency and to establish our future experiments in the field of space biology. From the biological point of view, we tried to pinpoint if the absence of gravity can be associated with the acquisition of microgravity-related phenotypes both in morphology and at molecular level. Instead, from a mechanical point of view, the RPM was tested to verify the characteristics of the machine, which best simulates a microgravity environment for cell cultures. To this end, two hardware devices were prototyped to evaluate (i) the number of revolutions of the RPM rotating arm and (ii) the acceleration undergone by the cell positioned on the RPM rotating arm.

## 2. Materials and Methods

### 2.1. Biological Characterization

#### 2.1.1. Cell Cultures and Microgravity Simulation Setup

The human TNBC cell line, MDA-MB-231, was purchased from the American Type Culture Collection (ATCC, Manassas, VA) and cultured within a cell incubator at a temperature of 37 °C and a CO_2_ concentration of 5%, in their proper culture medium as previously described [21]. One day before 0 g simulations, 4–5 × 10^5^ cells were seeded in a T25 tissue culture flask and stored inside the cell incubator in order to reach the confluence of 70% and to undergo microgravity the day after. Samples were prepared with the following configuration:

- Two T25 flasks to evaluate microgravity effects at the time point of 24 h after random positioning rotation;
- Two T25 flasks to evaluate microgravity effects at the time point of 72 h after random positioning rotation;
- Two T25 flasks as control (one for 24 h and one for 72 h) to be kept inside the incubator at the reference terrestrial gravity of 1 g.

Just before the beginning of the experiment, flasks were totally filled with complete cell culture medium, avoiding the presence of air bubbles to limit shear stress. T25 flasks were placed at the center of the RPM’s frame to minimize variations of the microgravitational state during the experiment. Finally, the RPM was stored within the cell incubator for the entire duration of the experiments (72 h). For the microgravity simulation setup and for other downstream investigations, three independent experiments were performed.

#### 2.1.2. Sample Collection

The s-μg allowed for discriminating among two distinct cell populations: the adherent one (AD) and the multicellular spheroid component (MCS). Separation and isolation of the two cell components were previously described by Hybel et al. [22]. Briefly, at the end of each time point of 24 and 72 h, T25 flasks were left inside the incubator vertically to allow the deposition of MCS on the bottom. The upper part of the culture medium (~20 mL) was gently aspirated, aliquoted, and stored at −20 °C for further analysis. The remaining cell culture medium, containing MCS, was aspirated and collected in tubes for centrifugation at 3000 rotations per minute (956 g-force) for 10 min. Isolated MCS were resuspended and washed in phosphate-buffered saline (PBS Gibco™, Paisley, Scotland), centrifuged at 3000 rotations per minute (956 g-force) for 10 min, the supernatant discarded, and the pellet stored at −80 °C for RNA extraction. For AD collection, T25 flasks were washed twice in PBS, cells were scraped and transferred into tubes to undergo the same centrifugation and isolation procedure followed for MCS. Control samples were collected using the same method as the AD component. Prior to cell isolation, each sample was photographed using a Leica DM IRB microscope (Leica Microsystems, Buccinasco, Milano, Italy) and cells were counted by a Burker camera before centrifugation and pellet recovery.

##### MTT Cell Viability Assay

To evaluate viable and metabolically active cells, 3-(4,5-dimethylthiazol-2-yl)-2,5-diphenyltetrazolium bromide (MTT) was used as previously described [23], with minor modifications. Cells were counted using a Burker counting chamber and seeded in 96-well plates at a final density of 10,000 cells/well (according to the manufacturer’s instructions) and MTT was added at a final concentration of 1 mg/mL to each well and incubated for 3 h under standard culture conditions. Then, the media of each well were then gently removed and 200 µL dimethyl sulfoxide (DMSO) was added to solve formazan crystals deposition. Plates were stirred on an orbital shaker for 10 min at room temperature. The absorbance was measured using a Multiskan SkyHigh Microplate spectrophotometer (Thermo Scientific, Milan, Italy) at 570 nm. Results were expressed as the percentage of MTT reduction versus control samples (control at 24 and 72 h, respectively).

#### 2.1.3. RNA Extraction and Quantitative RT-PCR Analysis

Total RNA was extracted from the MDA-MB-231 cells using the miRNeasy Kit (Qiagen, Hilden, Germany and RNA concentration was further estimated by spectrophotometry. Retrotranscription of the isolated RNA in cDNA was performed with the SuperscriptTM II Reverse Transcriptase (Invitrogen, Carlsbad, CA, USA); 1 μg of RNA was used according to the manufacturer’s instruction together with 250 ng of random primers in a final volume of 20 μL.

The primer sequences (forward and reverse), listed in Table 1, were chosen by using the Primer3 tool (http//fokker.wi.mit.edu/primer3) and the NCBI database.

Quantitative RT-PCR (qRT-PCR) analysis was performed in a final volume of 20 μL with the Fast SYBR™ Green Master Mix (Applied Biosystems, Waltham, MA, USA), using 20 ng of cDNA per each reaction, which was carried out in triplicate and with the following thermal cycle conditions: stage 1: 20 s at 95 °C; stage 2: (40 cycles) 3 s at 95 °C, and 30 s at 60 °C; stage 3: 15 s at 95 °C, 60 s at 60 °C, 15 s at 95 °C, and 15 s at 60 °C. After qRT-PCR, analysis of the melting curves was undertaken to verify the specificity of the reaction. Finally, data obtained were normalized referring to the housekeeping gene rRNA 18 s; each gene was amplified in triplicate and the average Ct value (cycle threshold) was analyzed with the 2^−ΔΔct^ method using SDS software (Applied Biosystems). Results are relative to the mRNA levels expressed in control samples grown for 24 and 72 h at the reference terrestrial gravity of 1 g. Results are expressed as mean value ± standard deviation (SD) of three independent experiments.

### 2.2. Mechanical Characterization

The purpose of the mechanical characterization was to verify whether the data provided by the manufacturer simulate an environment of microgravity for the cells placed in culture. Considering this, we evaluated (i) the number of rotations per minute that the rotating arm of the RPM performs and (ii) the acceleration undergone by the cell positioned on the rotating arm of the RPM.

#### 2.2.1. Rotations Measuring Device

**Figure 2:**
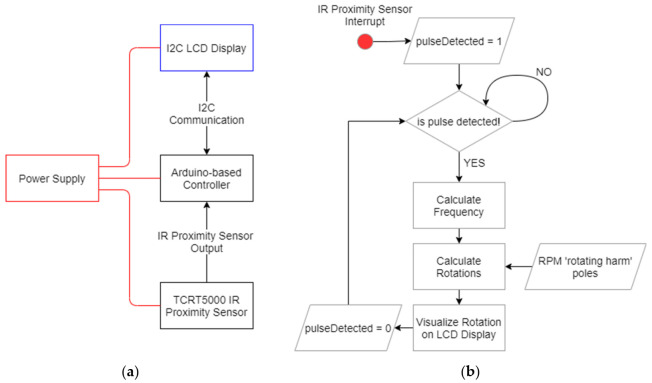
(**a**) Architectural and (**b**) functional schemes of the hardware device implemented for rotation measurement.

To measure and verify the rotations per minute performed by the RPM, a hardware reprogrammable device was implemented using an Arduino^®^-based microcontroller board. Figure 2a shows the architectural scheme of the hardware device. An infrared (IR) proximity sensor, equipped with a TCRT5000 emitter–receiver LEDs couple, was used to detect the movement of the rotating arm of the RPM.

The main processing unit is made by the Arduino^®^-based (Arduino Headquarters, Somerville, MA, USA) board which, upon receiving the output from the proximity sensor, calculates the rotation frequency and subsequently the rotation number, according to Equations (1) and (2):

(1) $$frequency=\frac{1}{∆T}\;$$

(2) rotations=poles×frequency 

where:

- $∆T=T_{1}-T_{2}$ represents the time elapsed between two consecutive reflections (output signals) received by the TCRT5000 (Chipskey Technology Co., Shenzhen, Guangdong, China) proximity sensor;
- poles is the number of times the rotating part of the RPM is detected at each complete rotation (in our case *poles* = 2).

Figure 2b depicts the functional scheme of the algorithm used to calculate the number of rotations.

#### 2.2.2. Acceleration Measuring Device

**Figure 3:**
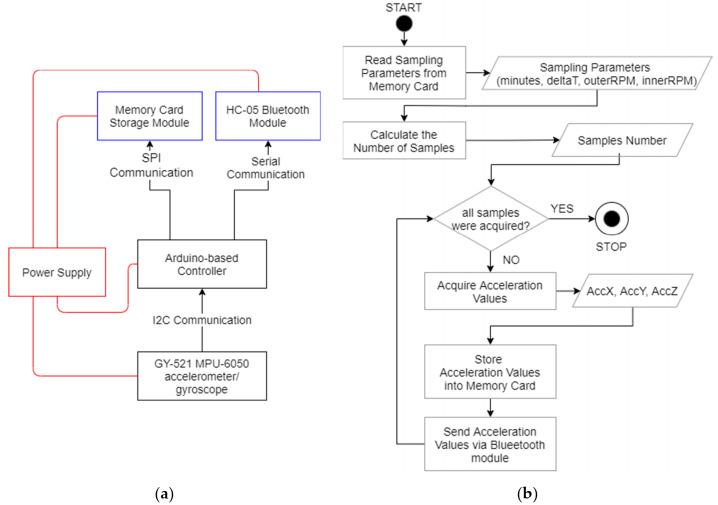
(**a**) Architectural and (**b**) functional schemes of the hardware device implemented for acceleration measuring.

The device to measure the acceleration undergone by the cells uses the GY-521 MPU-6050 module, equipped with a three-axis accelerometer and gyroscope. The management of the entire acquisition process is entrusted to a specially programmed Arduino^®^-based controller, which takes care of receiving the acceleration values by the GY-521 (three values along the x, y, and z axes) and sending them to the memory card module for storage and to the HC-05 Bluetooth module. Figure 3a,b shows the architectural and functional schemes of the implemented hardware device and its control algorithm used to calculate the acceleration values. Considering that the device does not have any type of ”wired” connection with the external environment—as it is positioned on the rotating arm of the RPM—the wireless communication provided by Bluetooth allows for real-time measurement feedback to a connected external device (e.g., a cell phone). Before starting the sampling, a setting file (previously written in the memory card) is read to determine the following two parameters:

- Duration of the sampling (in minutes)—*minutes*;
- Sampling interval (in ms)—*deltaT*.

From the values of *minutes* and *deltaT*, the number of samples to be acquired is calculated according to Equation (3):

(3) $$samples=minutes\times 60\times \frac{1000}{deltaT}\;$$

## 3. Results

### 3.1. Biological Characterization Findings

#### 3.1.1. Microgravity Simulations Drive Multicellular Spheroids Formation

**Figure 4:**
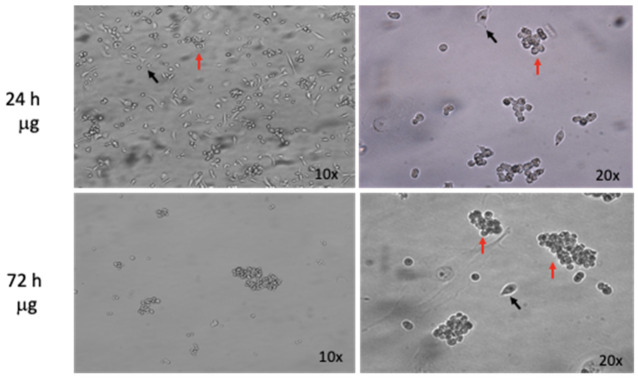
Simulated microgravity-induced formation of multicellular spheroids, MCS (red arrows) at 24 and 72 h in MDA-MB-213 cell line; few scattered adherent cells (AD, black arrows). Original magnification 10× and 20×.

As previously reported, when cultured in s-μg, cells form 3D-spheroids autonomously [24], hence, the first RPM biological characterization went through the evaluation of cell-spheroids formation at different time points, 24 and 72 h. The human triple-negative breast cancer cell line MDA-MB-231 was seeded 24 h before s-μg as described in the materials and methods section. When the specific experimental time point was reached, rotation was stopped and before cell collection each sample was photographed to detect 3D-spheroid formation. Figure 4 shows the formation of 3D structures at 24 and 72 h. Finally, we identified two different populations throughout the experiment: (i) adherent (black arrows) and (ii) floating (red arrows) with multicellular characteristics. The adherent fraction, called AD, was less represented relative to the floating and multicellular one, indicated as MCS, at the time point of 72 h.

#### 3.1.2. Cell Viability Is Differentially Affected by Simulated Microgravity

**Figure 5:**
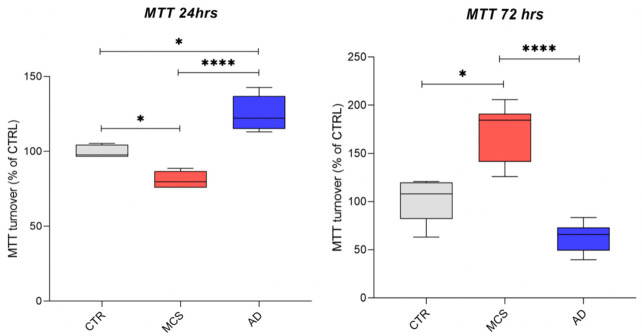
Assessment of cell metabolic activity by means of MTT Cell Viability Assay for AD and the MCS fractions of MDA-MB-231 cell line after 24 and 72 h of microgravity exposure. Data are represented via standard box and whisker plot of *n* = 3 independent experiments. * *p*-value < 0.05; **** *p*-value < 0.0001 (nonparametric Kruskal–Wallis test).

To assess a direct biological impact of s-μg on the MDA-MB-231 cells, we evaluated cell viability, one of the first aspects that is affected when cells undergo altered growth conditions. In our study, we sought to determine and compare the viability levels of the two different cell populations, isolated after RPM’s rotation at 24 and 72 h, respectively, by means of the MTT assay, comparing them with those of control cells grown at the reference gravity of 1 g. As shown in Figure 5, while the same level of cell viability is preserved in controls, MCS population undergoes an initial decrease at 24 h, followed by an increase in viable cells at 72 h. The opposite trend was evaluated in the AD cell fraction. A possible explanation of the results regarding the MCS population may rely on the physiological need of MDA-MB-231 cells to become accustomed to the new growing condition, which is characterized by a preliminary phase of diminished viability, followed by an increased proliferation after adaptation to microgravity. On the contrary, the AD fraction which persists in its ”normal” growing asset, goes from a level of viability similar to controls towards the cell death phenomenon due to their inability to adapt to gravity unloading.

#### 3.1.3. Gravity Unloading Influences Cell Behavior and Gene Expression

**Figure 6:**
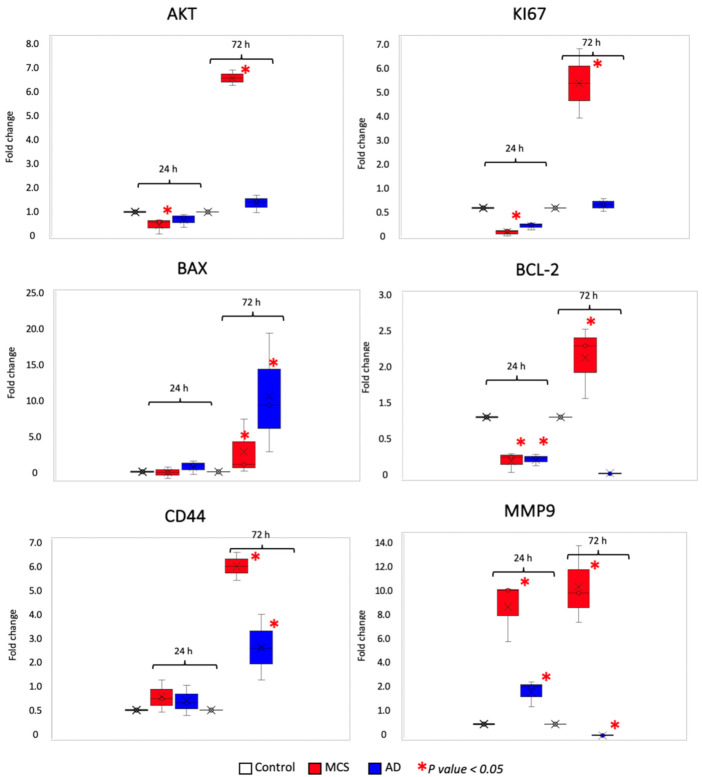
Results of qRT-PCR for the following genes and main regulated processes: AKT and KI67 (cancer proliferation); BAX and BCL2 (apoptosis); CD44 (cancer stemness), and MMP9 (metastasis). The data shown are relative to the mRNA levels in the MDA-MB-231 control cells at 24 and 72 h. Statistical analysis was performed using the nonparametric Kruskal–Wallis test (* *p* value < 0.05).

Since each single biological response or cell behavior is characterized by a change in gene expression, we aimed to highlight gene expression modifications induced by microgravity. Gene expression trends were analyzed by means of real-time PCR and different classes of genes were studied: two proliferation-related genes (AKT and KI67), two genes regulating cell death by apoptosis (BAX and BCL2), one gene related to cancer stemness (CD44), and another to cancer’s ability to migrate (MMP9). Gene expression results are grouped and illustrated in Figure 6.

Real-time PCR for proliferation markers was aimed at confirming the results obtained by the MTT assay comparing them with the expression of both AKT and KI67 mRNAs. Regarding the study of proliferation markers, the expression of both pAkt and KI67 did not undergo significant changes among all culture conditions analyzed, except for the MCS condition at 72 h. Indeed, consistent with the MTT assay, the only condition at which the AKT and Ki67 proliferation markers were significantly increased was the one referred to MCS population past 72 h from simulation.

We further investigated modifications in the anti- and pro-apoptotic balance to characterize cell death by apoptosis induced by microgravity. Apoptosis is a form of programmed cell death whose equilibrium is altered in cancer. We focused on two genes encoding two proteins that display two antagonistic roles: BAX and BCL2, BAX is a pro-apoptotic protein; conversely, BCL-2 promotes cell survival by inhibiting cell death [25]. Indeed, when the apoptosis equilibrium was investigated, the strongest increase of pro-apoptotic signals induced by BAX was highlighted in the AD populations at the time points of 72 h, suggesting that adherent cells chose to go towards a deathly fate. In the case of BCL2, playing an anti-apoptotic role, data are in line with the rationale behind BAX’s results interpretation. These results suggest that from the beginning of s-μg, cells probably enter a phase of uncertainty and need to adapt to the new environmental situation. Cells that overcome this phase start to proliferate, forming spheroids, the best spatial organization for growing when gravity becomes null. Conversely, cells that are unable to become accustomed to s-μg, as the AD population, enter a ”dormant phase” and eventually activate pro-apoptotic signals that lead them to death. Each of our results suggest a general tendency of MDA-MB-231 MCS to acquire a more aggressive behavior during s-μg. At the two experimental time points of 24 and 72 h, MCSs display an increase of both CD44 and MMP9 expression. While proliferation and survival markers may be considered as ”ordinary” features of tumor progression, related also to homeostasis phenomena, increasing levels of tumor stemness and capability to degrade the extracellular matrix are typical traits of tumor malignancy.

Our results show an overall and significant variation, both at 24 and 72 h, of the expression of genes analyzed.

### 3.2. Mechanical Characterization Findings

Concerning the verification of the number of rotations of the RPM rotating arm, 50 measurements were made. During these acquisitions, different combinations of *outerRotations* and *innerRotations* (controlling the rotation number of outer and inner RPM arms, respectively) were used. Considering that the ”nominal” value was set at 40 (*innerRotations* = 40), we obtained 39.95 ± 1.59 rotations per minute. Thus, we can say that the RPM actually performs the number of rotations set by the control display.

Instead, as for the measurement of acceleration, it was necessary to do a more in-depth analysis. Before the experimental measurements, the MPU-6050 was calibrated to set the offset values on the three axes and to allow the device to provide an initial measurement of 0 g in the static condition.

In particular, nine different acquisitions were made, each lasting 5 min, with a sampling frequency of 100 Hz. (This means a time step of 10 ms between two consecutive acquisitions). In fact, according to the Nyquist–Shannon theorem, the minimum sampling frequency necessary to avoid information loss must be greater than twice its maximum frequency. In our specific case—in the nominal setting (*innerRotations*, *outerRotations*) = (120, 40)—we have two periodic rotations with frequencies of 2 Hz (due to the 120 inner rotation) and 0.67 Hz (due to the 40 outer rotation). Considering the maximum frequency (2 Hz), an optimal sampling frequency must be greater than 4 Hz. Even in the worst case, which occurs with the setting (*innerRotations*, *outerRotations*) = (130, 50), double the maximum frequency is 4.34 Hz, and the value used for sampling is always valid. As a matter of fact, the frequency sampling of 100 Hz is sufficient to guarantee optimal signal sampling.

These nine acquisitions were obtained by considering all the combinations of the pair (*innerRotations*, *outerRotations*), obtained for *innerRotations* ∈ {110,120,130} and *outerRotations* ∈ {30,40,50}. In practice, we moved around the ”nominal” position (*innerRotations*, *outerRotations*) = (120, 40) of the RPM, which should guarantee 0 g. This RPM was able to operate in two different modes: (i) ”CONSTANT SPEED”, constant speed on a specific (inner or outer) rotation axis; and (ii) ”CONSTANT SPEED ALL”, constant speed on all rotation axes. According to the indications provided by the manufacturer, ”CONSTANT SPEED ALL” running mode was used.

Considering that the plate containing the cells has a nonpunctual dimension, in order to quantify the acceleration both in the central position of the rotating arm and in the lateral positions, for each of the nine combinations, three different positions of the measuring device were considered:

- In the center of the rotating arm (referred as “centered position”);
- Halfway between the center and the lateral position (referred as “half-lateral position”);
- In lateral position (referred as “full-lateral position”).

**Figure 7:**
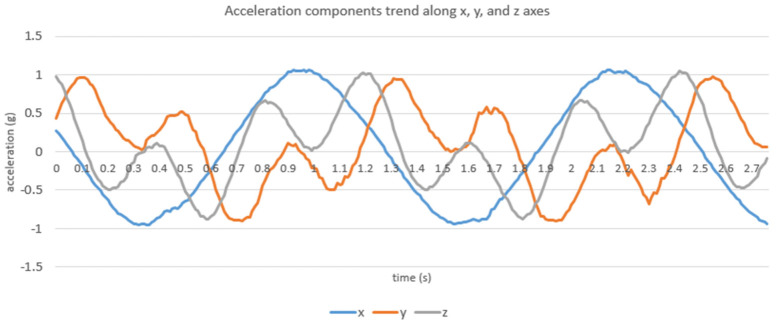
Example of the periodic trend of the acceleration components along the x, y, and z axes, obtained with the setting (*innerRotations*, *outerRotations* = (120, 40) in the ”centered” position (acquired with a 100 Hz sampling frequency).

The rotating arm of the RPM is 17.6 cm long and the acceleration device is 8 cm wide: this means that the useful space where the device can be positioned is only 4.8 cm (17.6/2–8/2 cm). In the ”half-lateral” and ”full-lateral” positions, the center of the acquisition device was positioned at 2.3 and 4.8 cm with respect to the center of the rotating arm, respectively. As expected, from the analysis of these data, it is possible to see that the three components along the x, y, and z axes have a periodic trend (Figure 7).

The RPM rotation causes the angular acceleration to counterbalance and cancel the natural g only in some parts of the rotation, when the directions of the two forces (g and acceleration due to rotation) are opposite. The period of acceleration components is correlated with the rotations per minute value; for example, in the case of (*innerRotations*, *outerRotations*) = (120, 40), we have a period of about 1.5 s. This means that every 1.5 s we measure both positive accelerations and negative accelerations, resulting in an almost zero acceleration undergone by the cells on average.

Table 2 reports the acceleration components values—in terms of mean value and SD—obtained in the “centered”, “half-lateral”, and “full-lateral” positions. Moreover, colored cells, with green, yellow, and light blue, highlight the three minimum averaged values obtained along the x, y, and z axes, respectively.

Analyzing the average values obtained for the three acceleration components, it is possible to see that they are very close to each other, with differences of the second or third decimal. In particular, it is possible to say that:

- For the “centered” configuration the setting (*innerRotations*, *outerRotations*) = (120, 40) could be fine;
- For the “half-lateral” configuration it is preferable to move toward (*innerRotations*, *outerRotations*) = (115, 45);
- For the “full-lateral” configuration the setting (*innerRotations*, *outerRotations*) = (120, 40) could be fine, even if the set of minima of the averaged values seems to move toward (*innerRotations*, *outerRotations*) = (115, 35).

## 4. Discussion

Humans have always been fascinated by the idea of visiting outer space. If the idea of exploring space was inconceivable at the beginning of the last century, however, the rush toward its conquest deeply accelerated the development of new technologies that brought animals first and men soon after in space by the end of the 1950s. The twenty-first century will witness the “coming-back” of humans to the Moon (planned for 2024), owing to the Artemis program, followed (hopefully soon) by the most important milestone of human history until today, the landing of a human crew on the Mars surface. On the other hand, space is one of the most inhospitable places to harbor human life, which is critically challenged by the space environment in many aspects of its physiology [3,4,5]. Besides the radiobiological concerns caused by the exposure to space radiations (e.g., Galactic Cosmic Rays and Solar Particle Events, GCR and SPE) [26], human health out of the Earth is jeopardized by the absence of the gravity force we experience in our daily life. Such a condition, known as microgravity, requires adaptation but also induces modifications that affect many aspects of human biology [15]. Although the presence of humans in space has been constant throughout the last 70 years, a very small number of individuals have had the chance to fly and experience life in space (almost 600). Hence, few clinical data are available to clarify the exact impact of microgravity on the human body [27]. Thus, tests focusing on the effects of gravity unloading can be performed on humans with the so-called “head-down tilt bed” studies [28,29], or can be more easily performed in vitro with the help of microgravity simulators [9]. 

Since human permanence in space will be in the future allegedly longer and longer, one of the main concerns will not be only about the management of acute effects induced by space life conditions, but also to develop countermeasures to handle chronic pathological conditions possibly affecting future space inhabitants. For this purpose, s-μg is applied in many field of research ranging from cell biology to physics [8,12].

Cancer still remains the second leading cause of death worldwide after cardiovascular diseases; thus, several studies have investigated the influence of microgravity on cancer cell growth, proliferation, stemness, and metastasis [30]. One of the most common cancers in women worldwide is represented by breast tumors, which are highly heterogeneous at the histopathological, molecular, and clinical level [31], and among these, the TNBC forms are one of the most aggressive and with a poor prognosis [20].

On these bases, we wanted to characterize mechanically the random positioning machine (RPM) and to investigate the biological effects of simulated microgravity (s-μg) on a TNBC cell line, the human MDA-MB-231, that we have used extensively as both in vitro and in vivo models for our previous studies [18,32].

Hence, the goal of our work was to collect data to evaluate the machine functioning for our future investigations in the field of microgravity simulations. To this aim, for the mechanical characterization, two hardware devices were developed to measure and evaluate (i) the number of revolutions that the rotating part of the RPM performs per minute and (ii) the acceleration undergone by the cell positioned on the RPM rotating arm. Regarding the measurement of the rotation number, by setting 40 rotations per minute, we obtained an average rotation number (on 50 measurements) of 39.95 ± 1.59 rotations per minute. Thus, we can assume that the RPM actually performs the number of rotations set by the control display. Instead, concerning the acceleration measurement, different combinations of *innerRotation* and *outerRotations* parameters were used (close to the couple (*innerRotation*, *outerRotations*) = (120, 40), which nominally should ensure 0 g). Even in this case, the acceleration undergone (simulated) at the center of the Random Positioning Machine rotating “arm” is on average 0 g. Overall, these experimental findings indicate that “nominal” values provided by the manufacturer represent, especially in the “centered” position, an optimal setting for our RPM to simulate μg.

For the biological characterization, we evaluated the status of some hallmarks of cancer that drive tumor progression, such as proliferation and survival, apoptosis, metastasis, and stemness [33]. We first investigated the change in morphology of MDA-MB-231 cells undergoing microgravity with respect to our control cells cultured in normal gravity conditions (1 g).

Cells in microgravity undergo a series of morphological changes that allow them to detach from their 2D growing surface and start growing in the liquid phase in a 3D fashion. In regular cell culture conditions, such as the one experienced on Earth, gravity attracts cells to deposit on the bottom of their support and forces them to expand, forming a cell layer. If cell layers are considered to be the regular way of culturing cells in cell biology, they do not resemble the physiological condition of cell growth in a human tissue. In fact, cells normally expand by exploiting each of the axes (x, y, and z) during tissue formation, and cell growth in vivo occurs through 3D structures called spheroids. Multicellular spheroids are currently used in basic research using scaffold-free or scaffold-dependent techniques [34,35] and represent a potent tool to mimic the in vivo microenvironment without resorting to animal models, used in many biological fields such as tumor modeling, tissue engineering, and drug testing [36]. In our experiments, indeed, we evaluated a change in morphology of MDA-MB-231 cells that underwent s-μg (after both 24 and 72 h) with respect to cells cultured at 1 g conditions. This result allowed us to prove the effective performance of our RPM device; moreover, it demonstrates that it is possible to use microgravity as a tool to create 3D cell structures in vitro and to alternatively investigate cancer biology from different perspectives with respect to 2D culture conditions.

Furthermore, since the adaptation to a new growing condition could impinge on cell proliferation and viability, we assessed cell metabolic activity by means of the MTT assay. 

Scientific literature reports that tumor cells exposed to microgravity are affected in terms of cell viability by enhancement of apoptosis; however, data still seems to be controversial, probably when different types of tumors are compared [37,38].

The microgravity condition allowed us to discriminate between two distinct cell populations arising during cell rotation: adherent (AD) and multicellular spheroids (MCS). In line with our adaptation hypothesis, the AD fraction showed a general decrease of cell viability; in fact, adherent growing does not represent the most favorable way to grow in microgravity. On the contrary, the MCS population increased cell viability at 72 h post s-μg, by time after a transient delay due to the adaptation to the new growing condition.

Each biological change is due to a modification of the gene expression profile. For this reason, we determined by real-time PCR if the expression of some genes related to survival, programmed cell death, cancer progression, and stemness, was affected by s-μg.

To study if gravity unloading is associated with cell proliferation and vitality, and to further confirm the survival data obtained by means of MTT assay, we analyzed the AKT and Ki67 genes.

The AKT gene encodes for a serine/threonine kinase that plays a crucial role in the Phosphatidyl Inositol 3-Kinase (PI3K) pathway. AKT is involved in a plethora of mechanisms that are often deregulated in tumors such as proliferation, migration, and cell survival, thus regulating many hallmarks of cancer. For its prominent role in tumor progression, AKT has been proposed as a therapeutic target [39], and it also plays a crucial role in TNBC, and its inhibitors have been tested in clinical trials and in combination with first-line chemotherapy for patients [40]. The nuclear antigen Ki67 is the proliferation marker by definition. It directly correlates with cell proliferation and its level of expression is used in diagnosis as a predictive and prognostic factor for TNBC [41].

In line with the MTT results, cell proliferation and viability, represented by AKT and KI67 gene expression, were more pronounced in the MCS collected at 72 h post s-μg; all the other conditions did not show any significant change.

Together with cell survival, we wanted to determine the expression of two genes that oppositely regulate apoptosis: BAX, which plays a pro-apoptotic function, and BCL2, which plays an anti-apoptotic role. Pro-apoptotic signals mediated by BAX were highlighted mainly in the AD population, especially at 72 h post s-μg. This result may be a direct sign of cell death as a consequence of cell survival instability in microgravity. The increase of BAX expression, also in the MCS fraction at 72 h, however, could be ascribable to physiological mechanisms of homeostasis, which contribute to spheroid development. Indeed, the generation of physical and chemical gradients of nutrients and oxygen occurs during spheroid formation. The core of the spheroid, generally characterized by a lower concentration of oxygen and nutrients, may be thus associated with apoptosis and a selective pressure to maintain only resistant and more aggressive viable cell fractions [42]. Overall, the BCL2 expression did not withstand any significant variation with respect to the controls and the other conditions, except for the MCS population at 72 h post s-μg, where the regulation of the balance between anti-apoptotic and pro-apoptotic signals seems to be more active.

Recently, the restoration of the BAX axis in TNBC has been demonstrated to hinder tumor progression [43]. On the contrary, the inhibition of the anti-apoptotic protein BCL-2 has been shown to increase tumor responsiveness to doxorubicin in TNBC [44]. Thus, the role of both BAX and BCL2 expression is pivotal in the TNBCs.

Finally, to assess if gravity unloading triggers a more aggressive phenotype in rotating samples with respect to cells grown in normal gravity conditions, we examined the gene expression levels of CD44 and the MMP-9 (Matrix Metalloproteinase-9). CD44 codes for a cell-surface antigen and its primary function is to act as the hyaluronic acid receptor. Despite its physiological role, CD44 is widely and highly expressed in several neoplasms and it participates in cancer cell proliferation, migration, and invasion [45]. Specifically, in the context of TNBCs, the expression of CD44 is linked to cancer stemness and linearly correlates with the amount of breast Cancer Stem Cells (CSCs) characterized by the CD44high/CD24low immunophenotype. CSCs represent a small population of cells within the tumor mass; however, they actively contribute to tumorigenesis, resistance to chemotherapy, and they are capable of self-renewal.

In TNBCs, the degree of cancer stemness is strictly associated with poor prognosis and cancer metastasis [46]. MMP-9 is a peptidase and its activity is to degrade the extracellular matrix and to induce tumor neovascularization during tumor progression and metastasis. The degradation of the matricellular components is one of the first steps to enable the extravasation of cancer cells toward secondary organs and thus to metastasize [47]. Hence, increasing levels of MMP-9 correlates with tumor aggressiveness and also have a prognostic relevance in TNBCs [48,49].

Each of our results suggest a general tendency of MDA-MB-231 MCS to acquire a more aggressive phenotype during s-μg. At the two experimental time points of 24 and 72 h, MCSs display an increase of both CD44 and MMP9 gene expression. While proliferation and survival markers may be considered as “ordinary” features of tumor progression, also related to homeostasis phenomena, increasing levels of tumor stemness and capability to degrade the extracellular matrix are typical traits of tumor malignancy. Surprisingly, in the case of the CD44 stemness gene, levels of expression also rise over time in AD at 24 and 72 h with respect to the controls; nonetheless, CD44 expression is more pronounced in MCS fraction at 24 h and especially at 72 h post s-μg with respect to all the other cell populations. In accordance, the MMP9 gene shows increased expression levels in MCSs at 24 and 72 h. These data suggest that once acquired a 3D structure, which closely resembles the natural organization of cells during tissue formation, a selective pressure within the spheroid fosters the acquisition of a more aggressive phenotype. In the AD fraction, except for a slight increase of MMP9 at 24 h, no significant results were obtained with respect to the controls.

Besides the mechanical verification of the RPM used in our studies, our biological findings highlight how s-μg impacts several biological features, including the change of expression of genes, which are actively involved in cancer progression.

When moving away from normal cell growth conditions, one of the most tangible effects of cellular impairment is characterized by changes in the proliferation rate. Together with proliferation, the disturbance of cytoskeleton polymerization is an indicator of cell wellness. For this purpose, several studies have examined the influence of s-μg in affecting such phenomena. Surprisingly, defects in cytoskeletal organization, which comprise both actin and microtubule polymerization, are not only ascribable to change in the magnitude and direction of gravity. Indeed, cytoskeleton formation can be affected by the mechanical stress deriving from cell organelles attached to cytoskeletal filaments and their change in weight during gravity unloading simulated by different devices such as a clinostat, RWV, or RPM [50].

When proliferation rate is analyzed, s-μg seems able to play opposite roles. In fact, s-μg can induce the nuclear localization of the Hippo pathway effector YAP in colon cancer cell lines [51]. YAP’s localization to the nucleus represents a critical step to induce cell proliferation [52] In contrast, other studies demonstrate that s-μg inhibits proliferation in other cancers such as lymphomas and gliomas [53,54]. However, the influence of s-μg on cell proliferation still remains an open debate [30] 

Previous works have also described a different trend in change of gene expression of different tumor cell-line models exposed to microgravity, including the MDA-MB-231 [55]. Thus, the application of s-μg not only could give us more insight concerning the evolution of carcinomas such as TNBCs, but hopefully may help us to unravel new molecular pathways and new biomarkers that are usually disguised in normal gravity conditions. Although the use of RPMs is widely diffused to experimentally recreate on Earth a condition that resembles microgravity, other groups have pointed out how biological samples can endure the effects of local forces and shear stress during rotation. These conditions may have an impact on cell behavior and should be further analyzed to highlight their role in the acquisition of a specific phenotype [56,57].

## 5. Conclusions

It is known that s-μg reproduces the effects that biological systems experience in a real space context. These effects can be traced back to the combination of many physical stressors that comprise acceleration forces, weightlessness, and adaptation to positive and negative pressure states that alternate over time. These effects affect the human body at its macroscopic level (organs and apparatus) and also at its microscopic levels (cells and tissue) [15]. Hence, microgravity, either real or simulated, represents a multifaceted topic that requires a deeper knowledge, including its role in cancer progression.

## Figures and Tables

**Table 1:** Primer sequences used for real-time PCR.

Gene Symbol	Forward Primer 5′> 3′	Reverse Primer 5′ > 3′
AKT	caaggacgggcacattaaga	gccgtagtcattgtcctcca
Ki67	cgtcccagtggaagagttgt	accccgctccttttgatagt
BAX	gaaccatcatgggctgga	cgtcccaaagtaggagag
Bcl2	ttgacagaggatcatgctgta	atctttatttcatgaggcacgtt
CD44	aacatggtccattcacct	agaggaagggtgtgctc
MMP9	gagaccggtgagctggata	tacacgcgagtgaaggtgag
RN18S1(rRNA18S)	cggacaggattgacagattga	agagtctcgttcgttatcgga

**Table 2:** Mean values and standard deviation of acceleration components with the device positioned in the “centered” position (upper section), “half-lateral” position (middle section), and “full-lateral” position (lower section). Colored cells, with green, yellow and light blue, highlight the three minimum averaged values obtained along the x, y, and z axes. All acceleration values reported are expressed in g units, while rotation values are expressed in rpm.

**“Centered” Position**											
**(inner, outer) = (110, 30)**				**(inner, outer) = (110, 40)**				**(inner, outer) = (110, 50)**			
	**accX**	**accY**	**accZ**		**accX**	**accY**	**accZ**		**accX**	**accY**	**accZ**
**mean**	0.0567	0.0256	0.0859	**mean**	0.0571	0.0207	0.0854	**mean**	0.0579	0.0228	0.0874
**SD**	0.5002	0.2679	0.2732	**SD**	0.4974	0.2709	0.275	**SD**	0.4977	0.269	0.2736
**(inner, outer) = (120, 30)**				**(inner, outer) = (120, 40)**				**(inner, outer) = (120, 50)**			
	**accX**	**accY**	**accZ**		**accX**	**accY**	**accZ**		**accX**	**accY**	**accZ**
**mean**	0.0499	0.0252	0.0877	**mean**	0.0565	0.0173	0.0838	**mean**	0.0639	0.0233	0.0869
**SD**	0.4995	0.2659	0.2718	**SD**	0.4797	0.2845	0.2723	**SD**	0.5011	0.2661	0.2707
**(inner, outer) = (130, 30)**				**(inner, outer) = (130, 40)**				**(inner, outer) = (130, 50)**			
	**accX**	**accY**	**accZ**		**accX**	**accY**	**accZ**		**accX**	**accY**	**accZ**
**mean**	0.0537	0.0316	0.0761	**mean**	0.0557	0.0229	0.0863	**mean**	0.0615	0.0236	0.0794
**SD**	0.4842	0.282	0.261	**SD**	0.4970	0.2621	0.272	**SD**	0.499	0.2629	0.2718
**“Half-Lateral” Position**											
**(inner, outer) = (110, 30)**				**(inner, outer) = (110, 40)**				**(inner, outer) = (110, 50)**			
	**accX**	**accY**	**accZ**		**accX**	**accY**	**accZ**		**accX**	**accY**	**accZ**
**mean**	0.1194	0.0152	0.0821	**mean**	0.098	0.008	0.0807	**mean**	0.0774	0.0091	0.0952
**SD**	0.5006	0.2626	0.2725	**SD**	0.4995	0.2644	0.2748	**SD**	0.5017	0.2626	0.2716
**(inner, outer) = (120, 30)**				**(inner, outer) = (120, 40)**				**(inner, outer) = (120, 50)**			
	**accX**	**accY**	**accZ**		**accX**	**accY**	**accZ**		**accX**	**accY**	**accZ**
**mean**	0.119	0.0104	0.0833	**mean**	0.0965	0.0086	0.0922	**mean**	0.0707	0.0067	0.0947
**SD**	0.5011	0.2646	0.273	**SD**	0.5058	0.2554	0.2749	**SD**	0.4998	0.2651	0.2732
**(inner, outer) = (130, 30)**				**(inner, outer) = (130, 40)**				**(inner, outer) = (130, 50)**			
	**accX**	**accY**	**accZ**		**accX**	**accY**	**accZ**		**accX**	**accY**	**accZ**
**mean**	0.1202	0.0105	0.0880	**mean**	0.0948	0.0089	0.0926	**mean**	0.0815	0.0021	0.0964
**SD**	0.4999	0.2628	0.2729	**SD**	0.4994	0.2611	0.2723	**SD**	0.4969	0.2655	0.2737
**“Full-Lateral” Position**											
**(inner, outer) = (110, 30)**				**(inner, outer) = (110, 40)**				**(inner, outer) = (110, 50)**			
	**accX**	**accY**	**accZ**		**accX**	**accY**	**accZ**		**accX**	**accY**	**accZ**
**mean**	0.0076	0.0167	0.0901	**mean**	−0.0181	0.0187	0.0895	**mean**	−0.0614	0.0242	0.089
**SD**	0.4973	0.2695	0.273	**SD**	0.4999	0.2682	0.2751	**SD**	0.4991	0.2666	0.2704
**(inner, outer) = (120, 30)**				**(inner, outer) = (120, 40)**				**(inner, outer) = (120, 50)**			
	**accX**	**accY**	**accZ**		**accX**	**accY**	**accZ**		**accX**	**accY**	**accZ**
**mean**	0.0071	0.0132	0.0923	**mean**	−0.018	0.0151	0.0898	**mean**	−0.0618	0.0202	0.0934
**SD**	0.4995	0.2718	0.271	**SD**	0.4961	0.2743	0.2700	**SD**	0.4976	0.2725	0.2729
**(inner, outer) = (130, 30)**				**(inner, outer) = (130, 40)**				**(inner, outer) = (130, 50)**			
	**accX**	**accY**	**accZ**		**accX**	**accY**	**accZ**		**accX**	**accY**	**accZ**
**mean**	0.0024	0.0182	0.0936	**mean**	−0.0216	0.0225	0.0918	**mean**	−0.0615	0.0199	0.0923
**SD**	0.4997	0.2667	0.2713	**SD**	0.4957	0.2679	0.2733	**SD**	0.4981	0.2654	0.271

## Data Availability

Not applicable.
